# Like father, like son

**DOI:** 10.7554/eLife.25669

**Published:** 2017-03-07

**Authors:** Claude Becker

**Affiliations:** Gregor Mendel Institute, Austrian Academy of Sciences, Vienna Biocenter (VBC), Vienna, Austriaclaude.becker@gmi.oeaw.ac.at

**Keywords:** paternal effects, epigenetics, substance abuse, Mouse

## Abstract

Exposing male mice to nicotine or cocaine enables their male offspring to cope with high doses of either, which suggests that such paternal effects are generic, rather than being a response to a specific type of stress.

**Related research article** Vallaster MP, Kukreja S, Bing XY, Ngolab J, Zhao-Shea R, Gardner PD, Tapper AR, Rando OJ. 2017. Paternal nicotine exposure alters hepatic xenobiotic metabolism in offspring. *eLife* **6**:e24771. doi: 10.7554/eLife.24771

Does the environment experienced by a parent have an influence on the physiological condition of its offspring? Despite much research, if and to what extent such influences exist remains a matter of debate (Heard and Martienssen, 2014). When discussing this question, we need to distinguish between epigenetic effects, which are expected to remain stable across several generations (and are thought to involve chemical modifications to DNA or histone proteins; Miska and Ferguson-Smith, 2016), and parental effects, which might only apply to first-generation offspring.

The environment experienced by the mother can affect the offspring in a number of ways – by, for example, altering the fitness of the unfertilized egg, or influencing the nutrition received by the fetus – and this complexity makes it difficult to determine how maternal effects influence the offspring (Wolf and Wade, 2009). It should be easier to understand how the environment experienced by the father affects offspring because, in mammals, fathers contribute little more than the sperm cell during fertilization (Crean and Bonduriansky, 2014). However, although several paternal effects have been reported (Rando, 2012), it has been difficult to tie a crucial event experienced by the father to a specific effect observed in the offspring. Now, in eLife, Oliver Rando, Andrew Tapper and colleagues at the University of Massachusetts Medical School – including Markus Vallaster as first author – report that many paternal effects (and maybe maternal effects as well) might be much more generic than previously thought (Vallaster et al., 2017).

In an elegant experiment, Vallaster et al. investigated how exposure of male mice to a toxin affects the offspring. For five weeks, male mice received drinking water containing nicotine. The mice were then deprived of nicotine for one week, to ensure nicotine was not present in their sperm fluid, before being allowed to mate with control females. In a control experiment, the fathers were kept on a nicotine-free diet before mating.

Contrary to expectations, the offspring in both groups were equally likely to survive after being injected with a single high-dose of nicotine (Figure 1A). Thus, exposing the fathers to nicotine did not appear to have an immediate "priming" effect on their offspring. However, the picture changed when Vallaster et al. ‘acclimated’ both groups of offspring to nicotine by adding small amounts of nicotine to their drinking water for several days. Among the acclimated mice, the chances of the offspring from nicotine-fed males of surviving the injection of a toxic dose of nicotine were almost twice those of the offspring of control males. Thus, a combination of supplying nicotine to the fathers with acclimation to low doses in the offspring leads to enhanced nicotine tolerance.

**Figure 1.**
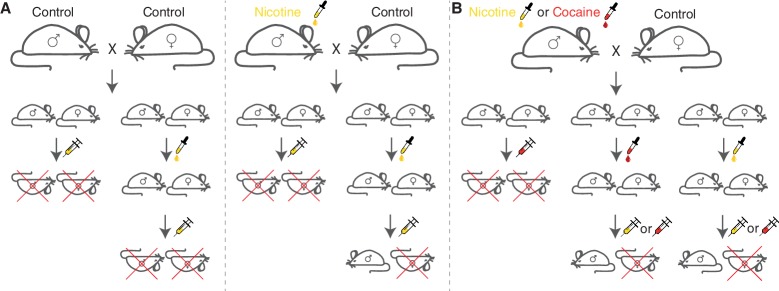
Generic paternal effects in the male offspring of mice. (**A**) Adult male mice were fed with normal drinking water (control mice; left) or drinking water containing a toxin (nicotine or cocaine; right), and then allowed to mate with control females. The offspring from control males and the offspring of males fed with nicotine are equally likely to survive the injection of a single high dose of nicotine (first and third columns). However, when they are acclimated to a chronic low-dose of nicotine, the nicotine-fed male offspring have a higher chance of surviving a high dose of nicotine (fourth column) than the offspring from control males (second column). Females, however, die in both groups. (**B**) The primed tolerance to toxins is non-specific: independent of whether males are fed nicotine or cocaine, their male offspring, after being acclimated to low doses of just one toxin, are more likely to survive a lethal dose of either toxin; mice that have not been acclimated are not likely to survive a lethal dose of either toxin.

In a search for a molecular explanation of this increased tolerance, Vallaster et al. found that the offspring of the nicotine-fed fathers had a higher detoxification rate of nicotine in their livers than the offspring of the control fathers. Related to this, a number of detoxification-related genes were also up-regulated in the offspring of the nicotine-fed fathers but not in the offspring of the control father. However, somewhat surprisingly, these genes were similarly up-regulated in both the acclimated and the non-acclimated offspring of the nicotine-fed fathers. It remains unclear why the increase in nicotine detoxification required exposure to nicotine in the father *and* in the offspring. A similar observation has been made in the model plant *Arabidopsis thaliana*, where increased tolerance towards saline conditions was achieved only after two subsequent low-dose treatments (Sani et al., 2013; Wibowo et al., 2016).

Vallaster et al. then checked if the effect was specific to nicotine. This involved exposing mice to either nicotine or cocaine before mating, and then acclimating their offspring to either the same toxin or the other toxin (Figure 1B). In all combinations where exposure of the father was followed by acclimation of the offspring, the detoxification capacity was higher. Even when fathers were simultaneously treated with nicotine and a drug that prevents nicotine dependence, the effect in the offspring persisted.

These findings show that repeated exposure to a specific toxin (nicotine or cocaine in this case) could cause a higher toxin tolerance in the offspring. This argues against a heritable acquired trait, which should be specific to the stress experienced by the parent. It will be interesting to see if these effects persist in a second or third generation of mice.

Intriguingly, only male offspring showed an increased drug tolerance after acclimation to the toxin. Further research is needed to investigate if this effect is linked to specific genes on the Y chromosome, to a process called X dosage compensation (in which genes on the X chromosomes in females behave differently to the same genes in males), or if there is another explanation. Lastly, studying the effects of nicotine on gene expression in the liver with higher cellular and temporal resolution should help us to ultimately understand the molecular mechanism underlying the potential to develop tolerance to toxins.
